# Gene deletion of P-Selectin and ICAM-1 does not inhibit neutrophil infiltration into peritoneal cavity following cecal ligation-puncture

**DOI:** 10.1186/1472-6890-4-2

**Published:** 2004-07-26

**Authors:** Elahé T Crockett, Crystal Remelius, Karen Hess, Hayma Al-Ghawi

**Affiliations:** 1 Departments of Physiology, College of Human Medicine, Michigan State University, East Lansing, MI, USA

**Keywords:** peritonitis, sepsis, transgenic mice, adhesion molecules

## Abstract

**Background:**

Neutrophil infiltration is one of the critical cellular components of an inflammatory response during peritonitis. The adhesion molecules, P-selectin and intercellular adhesion molecule (ICAM)-1, mediate neutrophil-endothelial cell interactions and the subsequent neutrophil transendothelial migration during the inflammatory response. Despite very strong preclinical data, recent clinical trials failed to show a protective effect of anti-adhesion therapy, suggesting that the length of injury might be a critical factor in neutrophil infiltration. Therefore, the objective of this study was to determine the role of P-selectin and ICAM-1 in neutrophil infiltration into the peritoneal cavity during early and late phases of peritonitis.

**Methods:**

Peritonitis was induced in both male wild-type and P-selectin/ICAM-1 double deficient (P/I null) mice by cecal ligation-puncture (CLP). Peripheral blood and peritoneal lavage were collected at 6 and 24 hours after CLP. The total leukocyte and neutrophil contents were determined, and neutrophils were identified with the aid of *in situ *immunohistochemical staining. Comparisons between groups were made by applying ANOVA and student t-test analysis.

**Results:**

CLP induced a severe inflammatory response associated with a significant leukopenia in both wild-type and P/I null mice. Additionally, CLP caused a significant neutrophil infiltration into the peritoneal cavity that was detected in both groups of mice. However, neutrophil infiltration in the P/I null mice at 6 hours of CLP was significantly lower than the corresponding wild-type mice, which reached a similar magnitude at 24 hours of CLP. In contrast, in peritonitis induced by intraperitoneal inoculation of 2% glycogen, no significant difference in neutrophil infiltration was observed between the P/I null and wild-type mice at 6 hours of peritonitis.

**Conclusions:**

The data suggest that alternative adhesion pathway(s) independent of P-selectin and ICAM-1 can participate in neutrophil migration during peritonitis and that the mode of stimuli and duration of the injury modulate the neutrophil infiltration.

## Background

Sepsis is a common cause of morbidity and mortality following surgery or trauma, and is characterized by activation of a systemic inflammatory response, severe hypotension, and major damage to multiple organs [1]. Although neutrophil migration into the tissue sites is crucial for effective elimination of infection, it also plays an important role in inflammatory tissue injury. The selectins, β2 integrins (i.e., CD18: Mac-1, LFA-1) and members of the immunoglobulin gene superfamily adhesion molecules, such as ICAM-1, play a significant role in neutrophil adhesion and transendothelial migration [2-4]. The expression and activation of these adhesion molecules on neutrophils and the endothelium, as well as the presence of a chemotactic gradient (eg. chemokines) appear to be important factors in neutrophil transmigration [3].

The selectins mediate neutrophil rolling while the β-2 integrins are important for firm adhesion and transendothelial migration [2-5]. The selectin family consists of three closely related cell surface molecules with differential expression by leukocytes (L-selectin), platelets (P-selectin), and vascular endothelium (P-selectin and E-selectin) [6]. ICAM-1 is one of the major ligands that binds to β-2 integrins (i.e., Mac-1 LFA-1) and is involved in neutrophil firm adhesion to endothelial and transendothelial migration [3]. ICAM-1 is constitutively expressed at a low concentration; however, under inflammatory conditions it is highly inducible in many cell types [7].

*In vivo*studies have shown that administration of small molecule ligands, and/or neutralizing antibodies to the selectins, ICAM-1 or β2-integrins can protect tissues from injury following endotoxin exposure, bacterial infection, or ischemia [8-10]. However, blocking reagents have the potential to stimulate or inhibit other receptors thus confounding the results. For example, a study by Kyriakides *et al*. has shown that soluble P-selectin attenuated skeletal muscle reperfusion injury by inhibition of the classical complement pathway [11]. Genetically altered mice deficient in adhesion molecules have been developed, which provide an alternative approach to study the role of the adhesion molecules in neutrophil recruitment and tissue injury. Recent studies using the transgenic mice have shown results indicating that neutrophils can use different adhesion pathways to emigrate from the systemic vasculature into the tissues and that the inflammatory responses may be site specific and stimulus dependent. For example, Mizgerd *et al*. have demonstrated a significant reduction in neutrophil infiltration into the peritoneal cavity at 4 hours of streptoccocal peritonitis in ICAM-1 mutant mice [8]. Another study by Kamochi *et al*. has shown that P-selectin and ICAM-1 significantly contributed to liver and lung injury at 4 hours of systemic endotoxemia in ICAM-1 and P-selectin/ICAM-1 double mutant mice [12]. Further, Bullard *et al*. have shown that P-selectin and ICAM-1 double mutant mice exhibited complete loss of neutrophil migration into the peritoneum during *S. pneumoniae*-induced peritonitis [13]. In contrast to these studies, Serman *et al*. have shown no differences in survival between wild type and ICAM-1-deficient mice following an intra-peritoneal injection with *E. coli*, *S. auerus*, or *P. aeruginosa *[14]. More importantly, recent clinical trials of anti-adhesion therapy in an attempt to reduce injury associated with traumatic shock and reperfusion injury failed to show a significant benefit despite strong preclinical data [15]. In an attempt to understand the disparity between the preclinical and clinical trial studies, it was noted that the lengths of injury in the clinical setting were longer than those of the preclinical studies. It appears that the underlying mechanism of neutrophil infiltration with a short period of insult is different from those of injury associated with a longer period of insult [15]. Additionally, adhesion-dependent and -independent neutrophil activation and migration can differentially be regulated by target tissue and mode of stimuli. Therefore, the goal of the study presented here was to investigate the role of ICAM-1 and P-selectin in peritonitis induced by CLP under a short and longer period of injury. The model of CLP-induced peritonitis used in this study is a clinically relevant model of sepsis [16]. This model was chosen to simulate the critical polymicrobial bacteremia-induced tissue injury that occurs in septic patients. The data of this study suggest that neutrophil infiltration into the peritoneal cavity following CLP can utilize an ICAM-1 and P-selectin independent pathway.

## Methods

All chemicals were purchased from Sigma Chemical (St. Louis, MO), unless otherwise noted.

### Animals

Only adult male mice (i.e., 8–10 wk) were used in this study. All animals received humane care in compliance with the *Guide for the Care and Use of Laboratory Animals *(National Institutes of Health Publication No. 85-23, revised 1985). Experimental protocols were reviewed and approved by the Michigan State University Animal Use and Care Committee.

Gene-targeted double mutant mice deficient in P-selectin and ICAM-1 (P/I double mutant), C57BL/6-Icam1^tm1Bay^Selp^tm1Bay^, backcrossed to C57BL/6, were used in this study. Breeding pairs of double-knockout mice were purchased directly from Jackson Laboratory (Bar Harbor, ME) and bred under the guidance of University Laboratory Animal Resources at Michigan State University. The wild-type (WT) mice were male C57BL/6, which were acclimated to the animal laboratory environment for one week before the start of experimentation. Before and after surgery, all the animals had unlimited access to food and water.

### Induction of peritonitis

#### Experimental model of polymicrobial sepsis

Polymicrobial sepsis was induced by CLP as previously described by Chaudry, *et al*. [16]. Surgical utensils were sterilized and all experimental procedures were performed under aseptic conditions. Adult male mice weighing 23 to 28 gm were anesthetized with inhaled methoxyflurane (Baxter Caribe Inc., Guayama, PR). The abdominal hair was shaved, the skin was cleaned with 75% ethanol using a sterile gauze and scrubbed with betadine solution. After drying, a 2-cm midline incision was made, the cecum was identified and ligated below the ileocecal valve using 0–5 silk suture with care being taken not to occlude the cecal valves. The cecum was punctured on both sides with a 21 G needle and gently squeezed to extrude a small amount of fecal material. The cecum was then restored to its normal position and the abdomen was closed in two layers using 5.0 nylon suture. Sham animals underwent the same procedures excluding ligation and puncture of the cecum.

#### Experimental model of peritonitis induced by 2% glycogen

Adult male mice (i.e., 8–10 wk) were lightly anesthetized by inhalation anesthesia with methoxyflurane. A volume of 2% sterile glycogen solution, equal to 10% of body weight, was injected intraperitoneally (i.p.). Six hours after glycogen inoculation, mice were euthanized and peritoneal lavage and blood samples were collected. The collected samples were identified with ID numbers to assure a blind fashion performance of the tests and data analysis. Total leukocyte and differential counts were performed as described below.

### Peripheral blood and tissue procurement

Blood samples were obtained from the right ventricle via a left anterior thoracotomy at the time of sacrifice, using a sterile heparinized syringe containing 50 μl of heparin (100 USP Units/ml). Blood smears were prepared and stained with Wright-stain (LeukoStat, Fisher Scientific, Pittsburgh, PA) for differential cell counts. The total number of peripheral blood leukocytes was determined by lysing the red blood cells using 3% acetic acid solution with the aid of a Neubauer hemocytometer. The remaining blood was centrifuged, and plasma was collected and stored at -70°C. A portion of the liver was fixed in buffered 10% formalin and embedded in paraffin, and a second portion was snap frozen in liquid nitrogen and stored at -70°C until used for immunohistochemistry staining.

### Collection of peritoneal lavage fluid

The peritoneal fluids were collected using repetitive (2 times) instillation and withdrawal of 2 and 5 ml respectively of sterile saline solution using a syringe with a 22 G needle. The peritoneal lavage sample was placed on ice, immediately processed for centrifugation at 4°C, and the supernatant and the cell pellet were collected separately. The cell pellet was used for total and differential cell counts. Differential cell counts were determined on cytospin preparations of peritoneal lavage stained with Wright-stain (LeukoStat). The total number of peritoneal leukocytes was determined as described above for the peripheral blood leukocyte count. Additionally, the presence of neutrophils in peritoneal lavage was confirmed by immunohistochemical staining of cytospin preparation using a primary antibody (IgG2a) specific to mouse neutrophil as described below. Further, the neutrophil content was quantified by measuring the myeloperoxidase (MPO) level of peritoneal lavage as described below.

### Demonstration of neutrophil recruitment by myeloperoxidase assay

The MPO contents of the peritoneal lavage supernatant and the cell pellet were quantified as previously published by our laboratory [17]. Briefly, the peritoneal cell pellet was resuspended in a potassium phosphate buffer, froze at -70°C, thawed, and sonicated for 40 seconds for two cycles (Ultrasonic Convertor, Model CL4, Misonix, Farmingdale, NY). The sample was then incubated at 60°C for 2 hours followed by centrifugation at 10,000 rpm for 5 minutes at 4°C. The supernatant was collected and used for the MPO assay. The MPO activity was determined using a tetramethylbenzidine substrate kit (ImmunoPure, Pierce, Rockford, IL) and read at 450 nm using a human leukocyte MPO as the standard. One unit of MPO activity was defined as the quantity of enzyme degrading 1 μmol peroxide/minute at 25°C. Similarly, the MPO content of peritoneal supernatant was measured.

### Determination of neutrophil infiltration and ICAM-1 expression by Immunohistochemistry

To confirm the identity of neutrophils in peritoneal lavage, immunohistochemical staining was performed using acetone-fixed cytospin cell preps. The primary antibody (clone 7/4, IgG2a) specific to mouse neutrophil (Cedarlane, Westbury, NY), the biotin-conjugated secondary antibody (PharMingen, San Diego, CA), and a Vectastain avidin-biotin complex reagent and 3,3'-diaminobenzidine chromogen kits (Vector Laboratories, Inc., Burlingame, CA) were used as previously described in detail [17]. The expression of ICAM-1 on endothelial cells was examined using acetone-fixed cryosections of the tissue. The liver was used in this study. Similarly, ICAM-1 expression was identified using a specific monoclonal antibody to mouse ICAM-1 (3E_2 _clone, PharmMingen, San Diego, CA) and a biotin-conjugated secondary antibody. Tissue sections were counter stained with hematoxylin (Gill's formula, Vector Laboratories) and mounted with DAKO Mounting Media (DAKO Corp, Carpinteria, CA). The samples were examined using a Nikon light microscope interfaced with a spot 24-Bit Digital Color Camera.

### Statistical analysis

All data were expressed as means ± standard error of the mean. Comparisons between two groups were performed using an unpaired *t*-test by way of *StatView version 5.0.1 software© *for Windows. Comparisons between multiple groups and various time points were analyzed using ANOVA with subsequent Fisher's PLSD test. *P *≤ 0.05 was considered significant.

## Results

### Verification of ICAM-1 and P-selectin deficiency in P/I null mice

**Figure 1:**
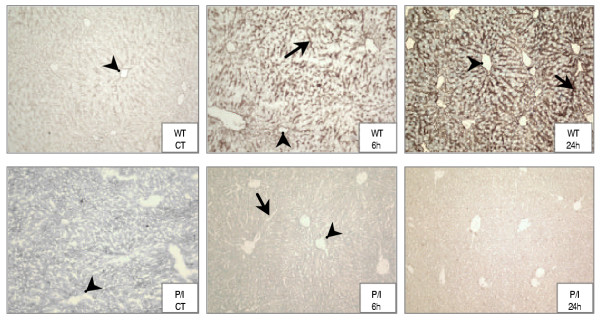
**Immunostaining of ICAM-1 expression in WT and P/I null mice. **Tissues ICAM-1 expression was determined by staining the liver sections with an anti-ICAM-1 antibody specific to mouse by applying the immunoperoxidase technique, and examined under a light NIKON microscope. The top row represents wild-type (WT) control (CT), CLP 6 h and CLP 24 h, respectively. Note the increased intensity of ICAM-1 staining of central veins (arrow heads) and sinusoids (arrows) with progression of sepsis. In contrast, ICAM-1 expression in P/I null mice liver sections was completely absent in controls, 6 h after CLP and 24 h after CLP (lower row). Control group represents mice that were not subjected to sham or CLP experimental treatment.

The Jackson Laboratory, where the P/I null breeding pairs were purchased, had initially tested the double knockout of the P/I null mice. In addition, the ICAM-1/P-selectin deficiency was confirmed in our laboratory in randomly selected litter mice tissue samples using RT-PCR and immunohistochemical staining of the liver tissue, as previously published by our laboratory (18). The ICAM-1 expression was determined in all the animals used in this study. Figure 1 shows ICAM-1 expression in liver tissues from the wild-type and P/I null mice by immunohistochemical staining technique. The ICAM-1 expression was constitutively present in wild-type control mice as indicated by light brown staining along the endothelium of the central vein, sinusoids, and portal vasculature (Figure 1, WT CT). The ICAM-1 expression was markedly increased in wild-type mice following CLP and continued to be evident at 24 hours of CLP (Figure 1, WT 6 h and WT 24 h). In contrast, ICAM-1 expression was absent in the tissues of P/I null mice before and after CLP treatment (Figure 1, P/I CT, 6 h and 24 h). The intestinal tissue ICAM-1 expression has also been examined, which was similar to that of the liver tissue. However, liver, due to its large endothelial cell content, serves as an excellent tissue source for ICAM-1 expression. For this reason, liver is routinely used in our studies to confirm the ICAM-1 expression.

### Clinical signs of sepsis

Clinical signs of sepsis were manifested as quietness, lack of response to stimulus, absence of socializing behavior, ruffled hair coat and lack of appetite. All mice subjected to CLP displayed these signs that became significant at 24 hours of CLP, and no striking differences were observed between the wild-type and P/I null mice.

### Effect of sepsis on leukocyte count in wild-type and P/I null mice

#### Peripheral blood leukocytes

To establish the blood cell parameters under normal physiological conditions in wild-type and P/I null mice, peripheral blood samples were collected from randomly selected mice. In this article these mice are denoted as "Control", which were not subjected to the sham or CLP operation. The leukocyte and differential counts of peripheral blood were measured. As shown in Table 1, the circulating leukocyte counts were not different between the wild-type and P/I null mice. However, the P/I null mice showed a significantly higher number of neutrophils and lower lymphocytes when compared to their wild-type counterparts (Table 1). There was no significant difference in monocyte count between the wild-type and P/I null mice.

**Table 1:** Leukocyte and differential counts in WT and P/I null mice. Randomly selected normal mice not subjected to the sham or CLP treatment, were anesthetized and the peripheral blood and peritoneal lavage collected for leukocyte content and differential analysis. Data are expressed as mean ± SEM. Total leukocytes for peripheral blood represents the absolute number of cells X 10^6^/ ml and for peritoneal lavage represent the absolute number of cells X 10^6^/ lavage. The values in parenthesis denote absolute number of the cells X 10^6^. * *p ≤ 0.05 *absolute number of cells of the wild-type compared to respective P/I null mice. *n *represents the number of mice per each group.

**Source**	**Total Leukocytes **(× 10^6^)	**Differential (%)**		
		
		**% Neutrophils**	**% Lymphocytes**	**% Monocytes**
**Peripheral blood**				
**WT **(*n = 4*)	10.4 ± 1.5	26 ± 2 (2.9 ± 0.5) *	72 ± 3 (7.2 ± 0.6) *	2 ± 1 (0.3 ± 0.1)
**P/I null **(*n = 5*)	10.3 ± 1.4	69 ± 7 (6.8 ± 0.9)	32 ± 6 (3.4 ± 0.5)	2 ± 1 (0.2 ± 0.1)
**Peritoneal Lavage**				
**WT **(*n = 6*)	3.8 ± 0.9	16 ± 1 (0.6 ± 0.2)	19 ± 3 (0.8 ± 0.3)	65 ± 5 (2.4 ± 0.7)
**P/I null **(*n = 6*)	3.6 ± 1.2	3 ± 1 (0.2 ± 0.1)	54 ± 5 (1.9 ± 0.4)	43 ± 6 (1.5 ± 0.7)

The induction of CLP-induced peritonitis resulted in a significant leukopenia in wild-type as well as P/I null mice as compared to their control counterparts (Table 2). It is interesting to note that although not statistically significant, leukopenia in the P/I null mice was less severe than those of the corresponding wild-type counterparts. This difference was mainly reflected by the presence of a higher number of neutrophils in the P/I null mice blood (Table 2). Similarly, CLP induced a significant neutropenia in both wild-type and P/I null mice when compared to the respective control mice. The most severe neutropenia occurred in the wild-type mice at 24 hours of CLP. In our studies peritonitis was also induced in response to 2% glycogen, which served as a positive control stimuli to induce an acute peritonitis as have previously been reported by many other investigators. As shown in Table 2, both wild-type and P/I null mice presented significant leukopenia at 6 hours of *i.p. *inoculation of 2% glycogen. Further, the inflammatory response to the sham operation, which elicited a trauma-induced peritonitis, caused leukopenia in both wild-type and P/I null mice. As shown in Table 2, there was a significant drop in the circulating blood leukocytes at 6 and 24 hours after the sham operation when compared to the respective control mice. However, a significant neutropenia was only present at 24 hours of the sham operation.

**Table 2:** Peripheral blood leukocyte and neutrophil counts in WT and P/I null mice subjected to CLP and 2% glycogen-induced peritonitis. *Control *group represents normal mice that were not subjected to sham or peritonitis treatment. *Sham (CLP) *represents the CLP respective sham group. Data are expressed as mean ± SEM, representing the absolute number of cells X 10^6^. * *p ≤ 0.05 *compared to respective control group; *# p ≤ 0.05 *wild-type compared to P/I null mice at the same time point and treatment. *n *represents the number of mice per each group.

**Experimental design**	**WT mice Total Leukocytes (× 10^6 ^/mL)**	**P/I null mice Total Leukocytes **(× 10^6 ^/mL)	**WT mice Neutrophils **(× 10^6 ^/mL)	**P/I null mice Neutrophils **(× 10^6 ^/mL)
**Control **(*n = 4*)	10.1 ± 1.3	10.3 ± 2.1	2.8 ± 0.7 **#**	6.7 ± 1.3
**Sham (CLP) 6 hr **(*n = 4*)	3.6 ± 0.6 *	4.5 ± 0.3 *	2.2 ± 0.1 **#**	3.8 ± 0.5
**Sham (CLP) 24 hr **(*n = 6*)	2.9 ± 0.7 *	4.4 ± 0.8 *	1.4 ± 0.3 *	2.3 ± 0.1 *
**CLP 6 hr **(*n = 6*)	2.0 ± 0.4 *	3.4 ± 0.9 *	1.2 ± 0.3 *	2.8 ± 0.9 *
**CLP 24 hr **(*n = 6*)	1.1 ± 0.3 *	3.8 ± 1.1 *	0.6 ± 0.2 *	2.8 ± 0.8 *
**2% Glycogen 6 hr **(*n = 4*)	3.4 ± 0.1 *	4.4 ± 0.8 *	2.5 ± 0.1	3.6± 0.6

#### Peritoneal leukocytes

Similar to peripheral blood, the cell parameters of peritoneal fluid under normal physiological conditions in the wild-type and P/I null mice were determined in randomly selected mice. The mice were not subjected to the sham or CLP operation. As Table 1 shows, no significant difference was found in total peritoneal leukocyte counts between the wild-type and P/I null groups (i.e., WT = 3.8 ± 0.9 × 10^6 ^vs. P/I = 3.6 ± 1.2 × 10^6^). However, it is interesting to note that monocytes/macrophages were the predominant cell type present in the peritoneal cavities of the wild-type mice, whereas in the P/I null mice lymphocytes were the predominant cell type (Table 1). Neutrophils constituted a smaller percentage of the peritoneal leukocytes in both the wild-type and P/I null mice (Table 1). Although the P/I null mice peritoneal lavage presented a lower percentage of neutrophils than those of the wild-type counterparts, the differences between the absolute numbers of neutrophil counts did not reach statistically significance (i.e., *p *= 0.08).

**Figure 2:**
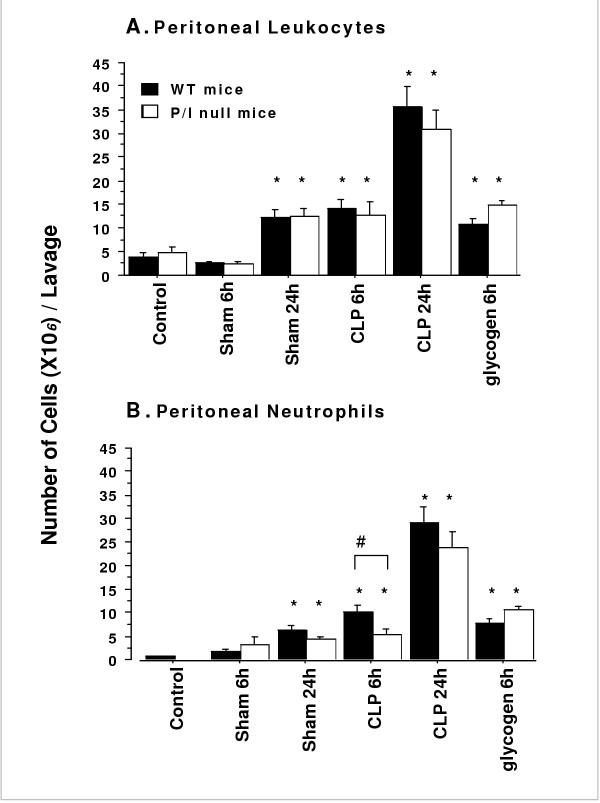
**Total peritoneal leukocyte and neutrophil counts in WT and P/I null mice subjected to CLP or 2% glycogen-induced peritonitis. **Wild-type (WT) and P/I null were subjected either to no treatment (*Control*), sham (*Sham CLP*), CLP, or 2% glycogen. The mice were then euthanized at 6, and 24 hours of treatment, peritoneal lavages were collected and analyzed for leukocyte and neutrophil contents. Data are expressed as mean ± SEM, representing the absolute number of cells X 10^6 ^per each lavage. Significant differences existed between sham, CLP and 2% glycogen as compared to their respective control group (* *p < 0.05)*, and as P/I null mice compared to respective wild-type at the same time period and treatment (*# p ≤ 0.05*). Data from six independent experiments with total sample size of 4 mice per each control and 2% glycogen group, 6 to 8 mice per each sham and 8 to 11 mice per each CLP treatment group.

**Figure 3:**
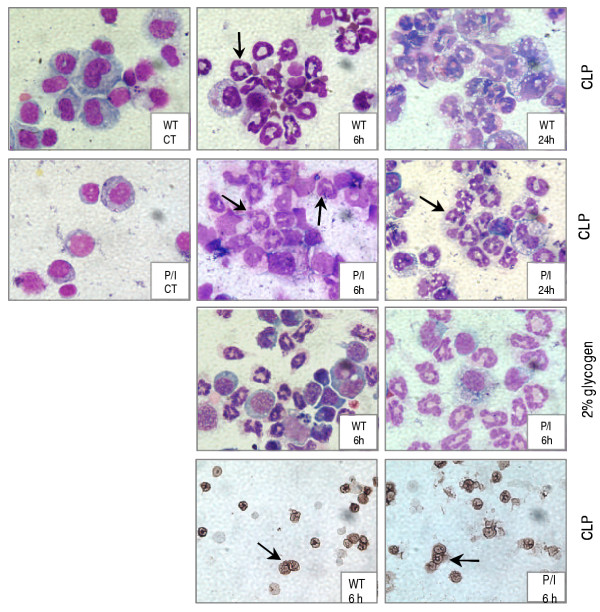
**Staining of cytospin preparations from peritoneal lavage. **The top three rows show the Wright staining of the cellular components of peritoneal lavages. Cellular preps from the control (CT) mice of both WT and P/I null groups demonstrating mononuclear cells as the predominant cell types (left column: WT CT, P/I CT). Control group represents mice that were not subjected to sham or CLP experimental procedures. CLP induced a significant neutrophil infiltration (arrows) at 6 and 24 hours in both WT (top row: WT 6 h, WT 24 h) and P/I null mice (second row: P/I 6 h, P/I 24 h). Neutrophils were also the predominant cell type at 6 hours of 2% glycogen-induced peritonitis in WT and P/I null mice (third row: WT 6 h, P/I 6 h). The lower row represents immunoperoxidase staining of the cells (CLP mice) using an anti-neutrophil antibody specific to mouse, verifying the neutrophils as stained in brown color (WT 6 h, P/I 6 h).

Sepsis induced by CLP (i.e., 6 and 24 hr) caused a significant leukocyte infiltration into the peritoneal cavities of both wild-type and P/I null mice when compared to their corresponding sham group (Figure 2A). The peritoneal leukocyte influx consisted predominantly of neutrophils as identified by Wright staining as well as in situ immunohistochemical staining using specific monoclonal antibody to mouse neutrophil (Figure 3). As shown in Figure 2B, a significantly greater number of neutrophils infiltrated into the peritoneal cavities of wild-type mice than those of the P/I null mice at 6 hours after CLP, which reached to comparable levels at 24 hours of CLP. Although a fewer number of neutrophils were present in the peritoneal cavities of P/I null mice, the ratio of neutrophil infiltration (i.e. ratio = infiltrated peritoneal neutrophils in response to CLP/neutrophils normally present in peritoneal cavity of the control mouse) was significantly higher in the P/I null mice than those of wild-type mice. There was a 16-fold and a 33-fold increase in the ratio of peritoneal neutrophils in wild-type at 6 and 24 hours after CLP, respectively. However, in P/I null mice, the peritoneal neutrophil infiltration ratio increased 54-fold and 204-fold at 6 and 24 hours after CLP, respectively.

Peritonitis was also examined in response to 2% glycogen. A significant leukocyte infiltration into the peritoneal cavity occurred in both wild-type and P/I groups at 6 hours after 2% glycogen injection (Figure 2A). Similar to the CLP response, the leukocyte influx into the peritoneal cavities in response to glycogen consisted predominantly of neutrophils (Figures 2B, 3). Further, sham operation caused a significant leukocyte infiltration into the peritoneal cavity of both wild-type and P/I null mice at 24 hours (Figures 2A, and 2B). Similarly, this peritoneal leukocyte influx consisted predominantly of neutrophils.

### Demonstration of peritoneal neutrophil recruitment by myeloperoxidase assay

**Figure 4:**
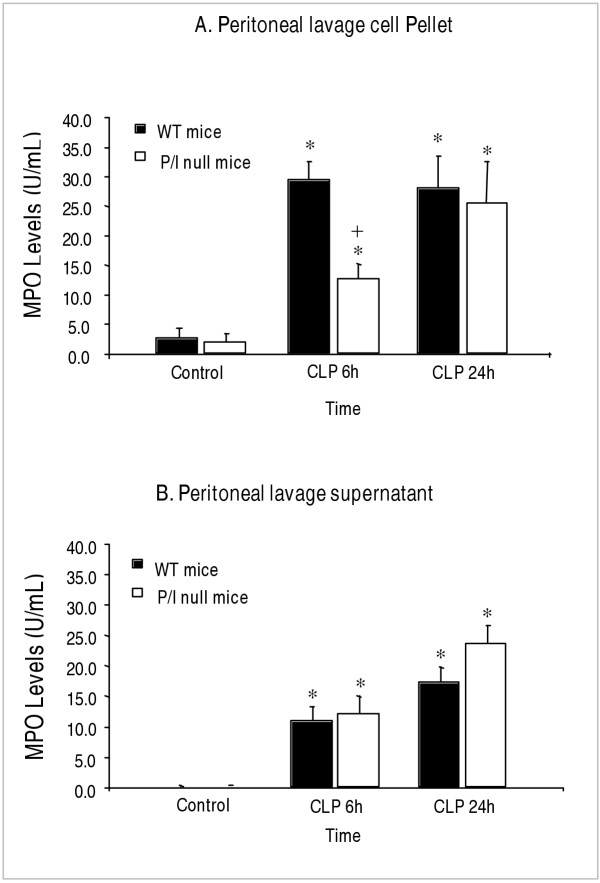
**Neutrophil infiltration as determined by MPO contents of peritoneal cell pellet and peritoneal supernatant. **Wild-type (WT) and P/I null were subjected to CLP, euthanized at specific time points, the peritoneal lavages harvested, centrifuged, and the supernatant and the cell pellet were collected separately and assayed for MPO contents. **(A) **Peritoneal lavage cell pellet. **(B) **Peritoneal lavage supernatant. Note that at 6 h after CLP, although a significant neutrophil infiltration into the peritoneal cavity of the P/I null group is present, it is significantly impaired compared to the corresponding WT group. Values are expressed as the mean ± SEM. * *p < 0.05 *compared to respective control group. + *p ≤ 0.05 *P/I null mice compared to respective wild-type at the same time period of CLP. Data from 6 independent experiments with total sample size of 8 mice per each treatment.

To further quantify the degree of neutrophil infiltration into the peritoneal cavity, the MPO levels of both the peritoneal lavage cell pellets and supernatants were measured. Although the MPO levels of the cell pellets indicated the presence of a significant number of neutrophils in the peritoneal cavity after CLP, the MPO values did not always correlate with the absolute number of infiltrated neutrophils. The Wright's staining of cytospin preparations of the peritoneal cells demonstrated highly activated phagocytes with vacuolized cytoplasm often containing numerous intracellular bacteria and in most cases with loss of cellular integrity (Figure 3). Thus, it was of concern to find out whether the MPO contents of neutrophils were released into the extracellular environment due to activation and loss of cellular integrity. To accomplish this objective, the MPO levels of the peritoneal lavage supernatants were also measured. As figure 4B shows, at 6 hours of CLP, significant levels of MPO were detected in the peritoneal lavage supernatants of both P/I null mice and wild-type animals when compared to those of the control mice. At 24 hours of CLP, further increases of MPO levels were present in the peritoneal supernatants of both mice groups. It is interesting to note that P/I null mice demonstrated a higher MPO levels in their peritoneal lavage supernatants than the wild-type counterparts. This increase may reflect a greater degree of neutrophils activation and/or loss of cell membrane integrity of the P/I null mice, and thereby the release of their MPO into the supernatant. The differences were not statistically significant.

### Demonstration of neutrophil recruitment by immunohistochemistry

Previous studies have indicated that tissue MPO activity can be affected by other factors (19, 20). and that MPO values may not indicate the true presence of neutrophils. Additionally, recent studies have identified MPO as the cellular component of macrophages (i.e., Kupffer cells) (21), as well as histiocytes (22). Therefore, an immunohistochemical staining technique was used to confirm the presence of neutrophils in the peritoneal cavity. The cytospin preparations of the peritoneal lavages were immunostained with a specific antibody to mouse neutrophil. Figure 3 (lower row) shows the Immunostaining of the peritoneal lavages obtained from wild-type and P/I null mice, in which neutrophils are stained in brown color.

## Discussion

Neutrophil infiltration is coordinated by the interplay of the adhesion molecules and the chemoattractants, which plays an important role in inflammatory tissue injury. Recent clinical trials of anti-adhesion therapy did not demonstrate a protective effect in trauma-induce shock despite very strong pre-clinical data (15). Further studies to clear this disparity suggested that the model systems applied and the length of injury are important factors that might modulate the underlying mechanism of neutrophil activation and migration (15). Therefore, this study was undertaken to examine the role of P-selectin and ICAM-1 molecules in the wild-type and P/I null mice subjected to a short and longer periods of peritonitis induce by CLP. The CLP model, chosen to mimic the normal course of sepsis in humans and animals, induced the classic signs of sepsis and a significant systemic inflammatory response. This response was associated with increased leukocyte infiltration into the peritoneal cavities of both the wild-type and P/I null mice. The leukocyte influx into the peritoneal cavity consisted predominantly of neutrophils (i.e., 62–80%), which significantly increased in both wild-type and P/I null mice at 6 and 24 hours of CLP. At the early phase of CLP (i.e., 6 h), the total number of neutrophils infiltrated into the peritoneal cavities of P/I null mice were significantly lower than those of the corresponding wild-type mice, which reached to a comparable level at 24 hours of CLP. In contrast to CLP-induced peritonitis, peritonitis induced by 2% glycogen exhibited no significant differences in the number of neutrophils infiltrated into the peritoneal cavities between the wild-type and those of the P/I null mice at 6 hours (Figure 2). The results of this study suggest differential regulation of the inflammatory response and neutrophil response by mode and length of the injury.

The data of this study showed that under normal physiologic environment the number of leukocytes present in peripheral blood and peritoneal lavage was comparable in the P/I null and wild-type mice. However, the ratio of neutrophils and lymphocytes were reversed between these two groups. In peripheral blood of P/I mice, neutrophils constituted a larger percentage of the leukocytes, which were significantly higher than those of the wild-type mice. In a striking contrast to the peripheral blood, the P/I null mice had a smaller number of neutrophils in their peritoneal cavities, when compared to those of the wild-type mice. Previous reports have also noted an increase in peripheral blood neutrophils in mutant mice [13]. However, to our knowledge, the decrease in peritoneal neutrophils has not been previously reported. It appears that within the normal physiologic environment, neutrophil trafficking in the peritoneal cavity is low in the P/I null mice and that neutrophil transmigration is regulated through adhesion pathways that utilize P-selectin and ICAM-1 molecules. The reason for increased circulating peripheral blood neutrophil counts in the P/I null is not known. However, this phenomenon has also been previously reported in CD18, P-sel/ICAM-1 mutant mice, and in patients with moderate or severe leukocyte adhesion deficiency [13,23,24]. Further investigation is needed to determine the following: whether hematopoiesis of neutrophils production is enhanced in the P/I null mice; removal of the neutrophils from blood circulation is reduced/delayed; and/or the neutrophil's life span has been increased.

In the present study, P-selectin/ICAM-1 appears to be partially involved in neutrophil migration into the peritoneal cavity but only during the early stages of the response to CLP-induced peritonitis. Conversely, these adhesion molecules were not required for maximal neutrophil migration after trauma (i.e. sham operation) or chemically-induced peritonitis (i.e 2% glycogen) and during the late phase of CLP (i.e, 24 hours). The data suggests that there might be a functional role for these adhesion molecules during the initial stages of the inflammatory response, and as the inflammatory process progresses, deficient adhesion mechanisms are bypassed. This notion has also been proposed in leukocyte recruitment in an *in vivo*experimental model of autoimmune encephalomyelitis, which is a T-cell-mediated disease. Kerfoot and Kubes have shown that during the early phase of encephalitis the leukocyte rolling was P-selectin dependent; however, with the progression of disease α4-integrin pathway was important in leukocyte rolling and adhesion [25].

Interaction of ICAM-1 expressed on the surface of vascular endothelial cells with the β_2_-integrins (eg., CD11b and CD18) expressed on neutrophils has shown to be a critical event mediating stable neutrophil adhesion and migration across the vascular endothelial barrier [26-28]. Although the study presented in this article suggests a non-role of ICAM-1 in neutrophil infiltration into the peritoneal cavity in response to peritonitis, it has to be noted that the P/I null mice are not a true ICAM-1 knockout. The P/I null mice may have a low level of alternatively spliced forms of ICAM-1 that could have been up-regulated on the vascular endothelium, and thereby promoting neutrophil migration [29]. Further, the lack of ICAM-1, *per se*, is not a critical factor that results in dysfunctional β_2_-integrin-mediated migration. Other adhesion molecule(s), ligand(s), and/or yet unknown counter-receptor(s) could mediate neutrophil infiltration. For example, ICAM-2, a ligand for β_2_-integrins, could be a potential candidate [32]. Other studies have shown a critical role of vascular cell adhesion molecule-1 (VCAM-1) in mediating neutrophil transendothelial migration and inflammatory tissue injury [30,31]. This adhesion molecule interacts with α4-integrin (α4β1, VLA-4). There is a body of evidence that α4-integrin can mediate several steps of leukocyte recruitment cascade (i.e., rolling and adhesion) through α4-integrin/MADCAM-1 (mucosal addressin cell adhesion molecule-1) and α4-integrin/VCAM-1 pathways. Vajkoczy et al., have shown that T-cells can adhere without rolling in spinal cord microvessels via α4-integrin [32]. Neutrophils express α4-integrin, and studies by Bowden et al. have demonstrated an important role of α4-integrin/VCAM-1 in CD18-independent neutrophil migration across mouse cardiac endothelium [33,34]. Additionally, the α4-integrin/VCAM-1-dependent neutrophil adhesion under flow conditions has been shown in neutrophils isolated from critically ill septic patients [35]. Neutrophil also express CD11d/CD18 and α9-integrin, which both bind VCAM-1, and could possibly, play an important role in neutrophil extravasation at sites of inflammation [36]. The importance of α4- and α9-integrin/VCAM-1 pathways in neutrophil infiltration in CLP-induced peritonitis remains unclear.

It is possible that neutrophil infiltration utilizes other secondary or tertiary adhesion pathways, and/or is facilitated by proteins that mediate the function(s) of the adhesion molecules. For example, a novel glycosylphosphatidyl inositol-anchored protein (GPI-80) that may regulate β_2_-integrin-mediated cell adhesion and motility of neutrophils has been described [38]. Additionally, other proteins are recognized to act as ligands for β_2 _integrins, such as those produced during coagulation as well as complement activation and tissue factor, which could facilitate neutrophil adhesion and infiltration into the peritoneal cavity [39,40]. Moreover, the local concentrations of chemokines appear to be critical factors in dictating the local neutrophil recruitment in an acute inflammatory response [41]. It has been shown that chemokines and their receptors are involved in leukocytes migration not only by inducing chemotaxis but also by regulating integrins to trigger cell arrest in shear flow [42].

Contrary to several other studies that have demonstrated the functional importance of P-selectin in models of myocardial infarction and inflammatory lung and liver injury, the data presented in this study indicated that P-selectin is not essential for neutrophil migration into the peritoneal cavity [43-45]. In support of our data, several studies have shown that blocking of P-selectin with monoclonal antibody or deletion of P-selectin and ICAM-1 did not inhibit neutrophil infiltration and tissue injury caused by hepatic reperfusion [18], endotoxin shock (46), i.p injection of 2% glycogen [47], and intestinal inflammation [48,49]. In contrast to our results of the P/I null mouse study presented in this article, Bullard et al., have previously reported a complete loss of neutrophil migration into the peritoneum of P/I null mice during peritonitis induced by *i.p. *inoculation of *Streptococcus pneumonia *[13]. The difference could due to the length of the experimental setting. In the Bullard *et al*. study, the inflammatory response and neutrophil infiltration into the peritoneal cavity was studied at 4 hours of the induction of peritonitis, while, in our study neutrophil infiltration was evaluated at 6 and 24 hours of peritonitis. In support of this reasoning is the study reported by Mizgerd *et al*. who have shown a compromised neutrophil migration into the peritoneal cavity of E-/P-selectin and ICAM-1 mutant mice in response to the injection of *Streptococcus pneumoniae *at 4 hours after injection, with no impairment present at 24 hours of bacteria injection [8]. Additionallt, Mizgerd *et al*. demonstrated that ICAM-1 is not necessary for neutrophil migration during glycogen-induced peritonitis, as shown otherwise by other authors [9]. Further, the difference could due to the possibility that in Bullard's study the infiltrated neutrophils into the peritoneal cavity of P/I null mice became necrotic and disintegrated upon challenges with *Streptococcus pneumonia *organisms. In our study, the data of peritoneal supernatant MPO levels have demonstrated a significant level of MPO released into the peritoneal cavity in mice subjected to CLP (Figure 4B). In Bullard's study the MPO levels of the peritoneal lavages were not measured. Further, in our study the Wright staining as well as the *in situ *immunohistochemical staining of the peritoneal cells clearly confirms the presence of neutrophils in the cytospin preparations (Figure 3). The data collectively indicate that P-selectin and ICAM-1 each may have a role in neutrophil migration during early stages of acute bacterial peritonitis, but at later stages alternative pathways are recruited to mediate neutrophil migration. Functional redundancy of adhesion molecules and the cytokine production may be sufficient to compensate for the absence of P-selectin and ICAM-1 in mediating neutrophil infiltration into peritoneal cavity in response to CLP in P/I null mice.

Another important difference between the results of the study presented in this article and those of previous studies may relate to the employment of essentially different models of sepsis and inflammation. While previous studies employed hemorrhagic shock-, *Streptococcus- *or LPS-induced peritonitis, the present study employed polymicrobial septic peritonitis as well as chemical and trauma-induced peritonitis. Published studies have shown quantitative and qualitative differences in the inflammatory response induced by gram-positive, gram-negative, polymicrobial sepsis and purified endotoxin [50-52]. These differences include: the species of bacteria employed and their respective antigens, the magnitude of leukocytic response, the magnitude and kinetics of cytokine production, and finally, mortality. Thus, the collective data suggest that the model of septic insult used to induce an inflammatory response is an important consideration. One major advantage of the model used in our study is that the relative magnitude of the inflammatory response in animals subjected to CLP-induced peritonitis is similar to those observed in septic patients [53,54].

## Conclusions

The data presented in this study have shown a clear, time-dependent neutrophil migration and infiltration into peritoneal cavity in responses to peritonitis, which is independent of P-selectin and ICAM-1 adhesion molecules. The negative nature of the data presented here and the failure of the anti-adhesion clinical trials, are of great importance. These findings demand innovative alternative approaches to neutrophil transmigration in inflammatory response and suggest that targets other than these adhesion molecules need to be identified. A better understanding of the mechanisms leading to neutrophil migration is critical for the development of new therapeutic strategies for treating inflammatory disease without compromising the host's immune response mechanisms.

## Competing interests

None declared.

## Authors' contribution

CR participated in the technical procedures for CLP, collection of blood and tissue samples, and MPO assay. KH and HA participated in preparation of tissue sample for immunohistochemistry and special staining. KH also assisted in preparation of the manuscript. EC conceived the study, and participated in its design, coordination, and preparation of the manuscript. All authors read and approved the final manuscript.

## Abbreviations

CLP, cecal ligation-puncture; ICAM-1, Intercellular adhesion molecule-1; mAbs, monoclonal antibodies; MPO, Myeloperoxidase; P/I null mice, P-selectin/ICAM-1-deficient mice

## Pre-publication history

The pre-publication history for this paper can be accessed here:


